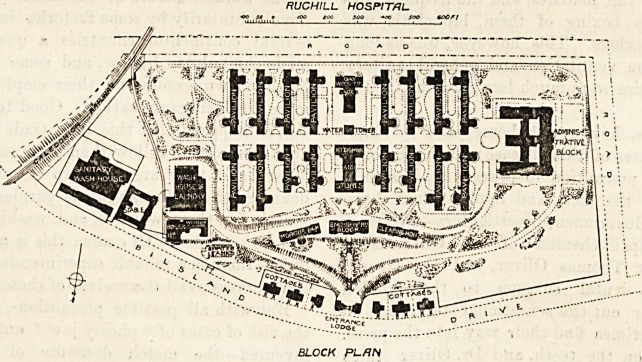# The Ruchill Hospital, Glasgow

**Published:** 1901-05-04

**Authors:** 


### The Institutional Workshop.

### THE RUCHILL HOSPITAL, GLASGOW.

For about thirty years the Corporation of Glasgow have had in contemplation the erection of a hospital for infectious diseases; but for a long time it was impossible to obtain a suitable site, there being no available space within the municipal boundaries. With the passing of the Glasgow Police Act of 1890, the Town Council obtained power of acquiring hospitals beyond the city, and in 1892 land was bought at Ruchill. Part of this land was to be devoted to a hospital for infectious diseases.

RUCHILL HOSPITAL. GLASGOW.

PLAN OF PAVILION.

M S McDonald

Architect

Glasgow.

The site was extremely well chosen, as not only is it on high ground ensuring plenty of pure air, and permitting of easy drainage, but it is readily accessible from various parts of the city.

There is only one entrance to the hospital grounds, and that is in Bilsland Drive. The entrance lodge is double, and on each side of it are three houses occupied by the house steward, the clerk of the works, and by other non-professional officers.

RUCHILL HOSPITAL

BLOCK PLAN

Altogether there are thirty-four blocks, of which sixteen are for the treatment of patients. Twelve of these have space for thirty beds each, and four have space for twenty beds each. This makes a total of 440 beds. The cubic space is ample, and gives 2,000 cubic feet to each bed.

It is evident that much consideration has been given to the planning of this hospital. The committee have availed themselves of the experience gained at the Belvedere Hospital, Glasgow, and they have also studied the arrangements in other infectious hospitals in various parts of the kingdom.

Passing through the entrance gates we find a road going eastwards and connecting the water-tanks, workshops, boiler-house, and laundry. A similar road on the west passes to the administrative block. Almost due south a footwalk leads to the dead-house, the inquiry office, and the clearing-house block. The patients' blocks lie between these and the high road. As already said, there are sixteen of these, and they are divided into two main groups by the kitchen and stores block, the water tower, and the day-workers' block.

The pavilions lie north and south. The wards are divided into two—and each of these is again divided into a convalescent ward of five beds and an acute ward of ten beds. Placed between these are a nurses' room and a pantry. At right angles to the wards is an adjunct consisting of a hall, having doors opening into the wards and to the grounds, a bath-room, a lavatory and closets, the latter being cut off from the rest by a cross-ventilated passage. Excepting as regards their size, the wards are all on the same plan.

The administrative block, placed westwards, is a building having a frontage of 240 feet. The component parts are arranged in the shape of the letter E. The block contains ample provision for the medical superintendent, the matron, for the whole of the professional staff, and for 200 nurses. In the event of non-infectious diseases appearing among the nursing staff, sick rooms are provided. There are sitting-rooms for the nurses, recreation rooms, bath-rooms, and telephone offices—indeed it would seem as if nothing had been forgotten. The dead-house is fitted up with laboratory, museum, lecture-hall, and post-mortem rooms.

The clearing-house has waiting-rooms, retiring-rooms, and six bath-rooms for men and six for women.

The water tower is so arranged that it provides storage of water on four different levels. In the day-workers' block are 78 bedrooms, recreation-room, bath-rooms, napery stores, &c. The style of architecture is Elizabethan, but freely treated, as required by the purposes for which the building is intended. Some of the blocks are built of Dumfriesshire freestone, others are of Scottish terra cotta brickwork and freestone facings.

The establishment is lighted by electricity, and warmed by open fire-places and hot-water pipes. The entire cost of the hospital was £250,000. Clearly expense has not been spared; but the hospital may claim to be "as perfect as thought and experience could devise."

The architect is Mr. A. B. Macdonald.

## Figures and Tables

**Figure f1:**
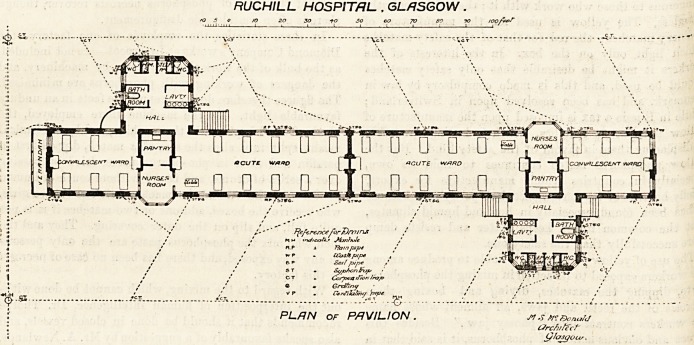


**Figure f2:**